# The clinical application of ear keloid using five-blade core excision combined with pressure and superficial electron beam radiation

**DOI:** 10.3389/fmed.2025.1634961

**Published:** 2025-10-22

**Authors:** Ying Qi, Suling Xu, Jieyi Wang, Chao Xin, Xiaohui Li, Bingjiang Lin

**Affiliations:** ^1^Department of Dermatology, The First Affiliated Hospital of Ningbo University, Ningbo, Zhejiang, China; ^2^Department of Laboratory Medicine, The First Affiliated Hospital of Ningbo University, Ningbo, Zhejiang, China; ^3^Department of Ophthalmology and Otorhinolaryngology, Ningbo Yinzhou No. 2 Hospital, Ningbo Urology and Nephrology Hospital, Ningbo, Zhejiang, China

**Keywords:** keloid, core excision, ear, superficial electron beam radiation, clinical application

## Abstract

**Background:**

Surgical removal is the primary method for the clinical treatment of ear keloids. However, there are numerous surgical options available, and no standardized approach in the literature.

**Objectives:**

This study aimed to evaluate the impact of five-blade core excision on the removal of ear keloids.

**Methods:**

A preliminary study involving 11 patients (21 lesions) with ear keloids was conducted between January 2023 and December 2023. Five-blade core excision was performed; superficial electron beam radiotherapy was administered at a dose of 4 Gy for 5 post-operative consecutive days, and pressure clips were applied for 6 months. The Vancouver Scar Scale (VSS) and the Patient and Observer Scar Assessments Scale (POSAS) were used to assess the results.

**Results:**

The mean age of the patients was 24.36 years (18–44 years). Postoperative follow-up ranged from 20 months. The patients underwent 5 days of postoperative radiotherapy and pressure clips for 6 months. Nine patients had no recurrence, whereas two patients had a mild recurrence (one patient rejected radiotherapy). The VSS and POSAS scores significantly decreased (*p* < 0.01).

**Conclusion:**

Five-blade core excision combined with pressure and superficial electron beam radiotherapy demonstrates effective therapeutic outcomes for ear keloid.

## 1 Introduction

The formation of a keloid, a benign skin tumor, is triggered by various factors such as trauma, infection, and burns (1). During the repair process, there is an abnormal proliferation and excessive deposition of fibroblasts, leading to the expansion of the wound surface beyond its origin (2, 3). The primary manifestation is a highly invasive red mass, and it cannot be resolved spontaneously. It commonly occurs in the anterior chest, shoulder, mandible, and ear (3, 4). The treatment methods for this condition encompass the application of topical medications, local injections, pressure therapy, surgical intervention, laser procedures, cryotherapy, radiation therapy, and so on (4–8). Due to the recurrent nature of keloids, there is no universally accepted standard treatment. Treatment options must be tailored to each case and comprehensive in approach (9).

The ear is a frequently affected site for keloid formation (8), primarily resulting from ear piercing, as well as other factors, including trauma, burns, and mosquito bites. The prevalence of keloid formation resulting from ear piercing is approximately 2.5%, with the highest occurrence observed in individuals aged 11 years and above (10–12).

The forms of ear keloids present in diverse forms, including single or multiple occurrences, located in front of or behind the ear, encompassing the entire earlobe (or ear rim), and even resulting in the complete loss of the outline of the ear. The current classification of ear keloids lacks uniformity. According to the literature review, there are Change-Park classifications and a classification based on anatomical location (13, 14), and the classification is primarily based on the choice of treatment.

The treatment of ear keloids can broadly be categorized into two main approaches: surgical interventions and non-surgical methods. The non-surgical methods include cryotherapy, intralesional injections, laser therapy, pressure dressings, and radiation therapy. Due to the short treatment period, favorable outcomes, and improved postoperative esthetics, surgical intervention is commonly selected as the primary approach for ear keloid management. Typically, this procedure necessitates adjunctive measures such as radiotherapy and local compression to minimize keloid recurrence (8, 15). The primary surgical techniques for treating ear keloids include direct excision and suturing, core incision, skin grafting, and various flap procedures (11, 13, 15–17). The core excision technique is commonly used in surgical interventions for ear keloids.

The present study discusses a straightforward and convenient approach to the core excision technique—the five-blade technique.

## 2 Methods

The study retrospectively collected 11 patients (with a total of 21 lesions) diagnosed with ear keloid at The First Affiliated Hospital of Ningbo University between January 2023 and December 2023, comprising 10 female and 1 male individuals. The age range of the patients was 18 to 44 years old, with a mean age of 24.36 years. The patients had a history of ear keloids for 1–8 years, mean of 1.33 years.

The study was approved by the Ethics Committee of The First Affiliated Hospital of Ningbo University (serial number 2024062RS-YJ01). The technique and use of photographs were performed with the written informed consent obtained from all patients.

### 2.1 Patient selection criteria

All patients were clinically diagnosed with auricular keloids.

The inclusion criteria were as follows:

1. Individuals with keloids persisting for ≥6 months, demonstrating progressive growth, erythema, induration, pruritus, and/or pain;
2. Those with lesions exhibiting tumor-like hyperplasia extending beyond the original wound margins, involving adjacent normal skin, with a characteristic tendency for eversion and site predilection;
3. Those whose complete medical records are available and have adequate cognitive/communication capacity; and
4. Those aged ≥18 years.

The exclusion criteria were as follows:

1. Individuals with a history of malignancy, active tuberculosis, immunodeficiency disorders, or other significant systemic comorbidities; and
2. Those who were pregnant, those who were lactating, or those with plans for pregnancy within the study period.

### 2.2 Operative technique

After routine disinfection, a 0.2% ropivacaine injection containing epinephrine at a concentration of 1:100,000 was administered to infiltrate the base of the keloid for anesthesia until sufficient anesthesia was achieved.

**Figure 1:**
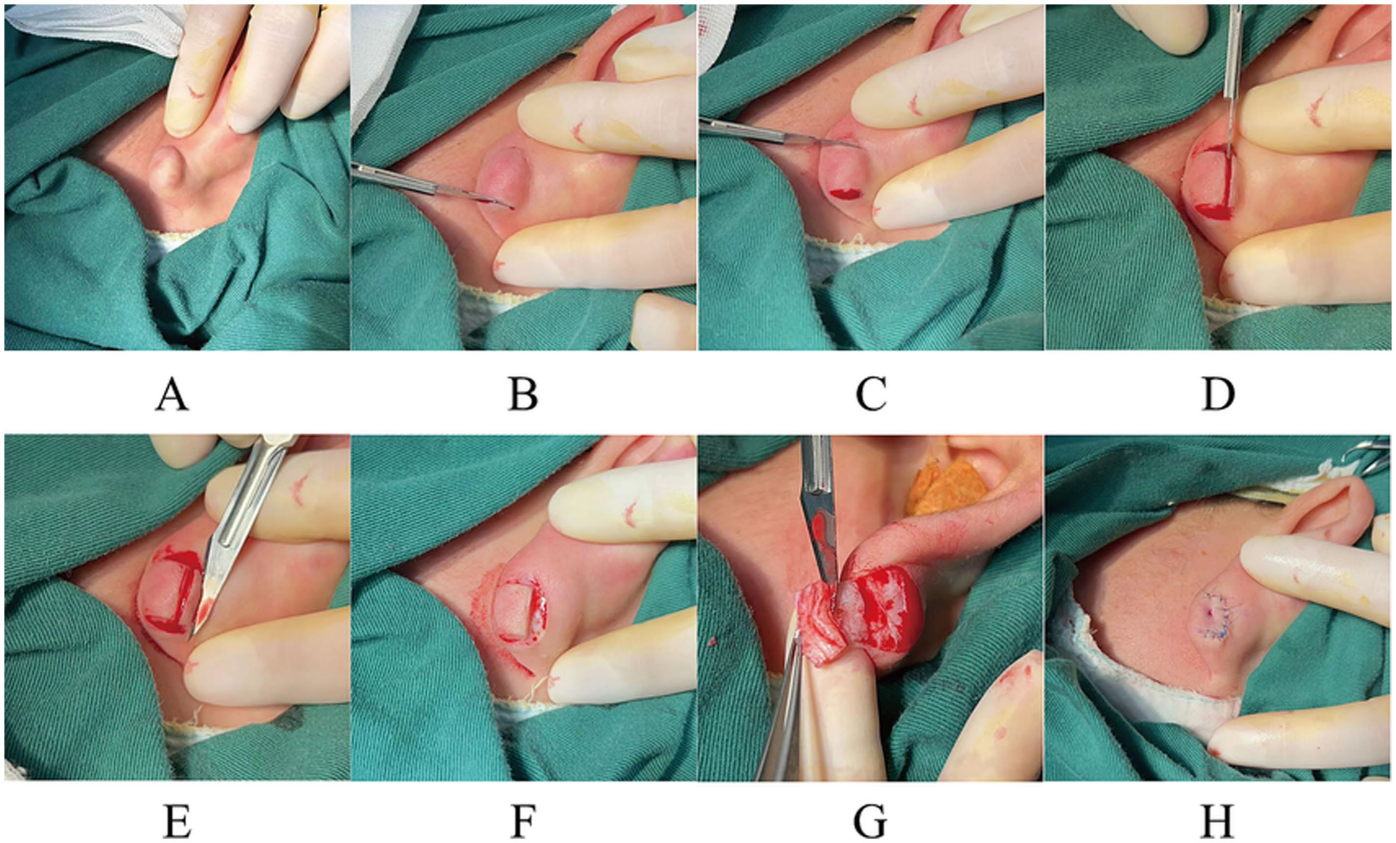
**(A,H)** Preoperative and postoperative views of a 22-year-old woman with a keloid on her left ear. **(B–E,G)** Course of the first to fifth blade core excision. **(F)** State after the fourth blade core excision.

**Figure 2:**
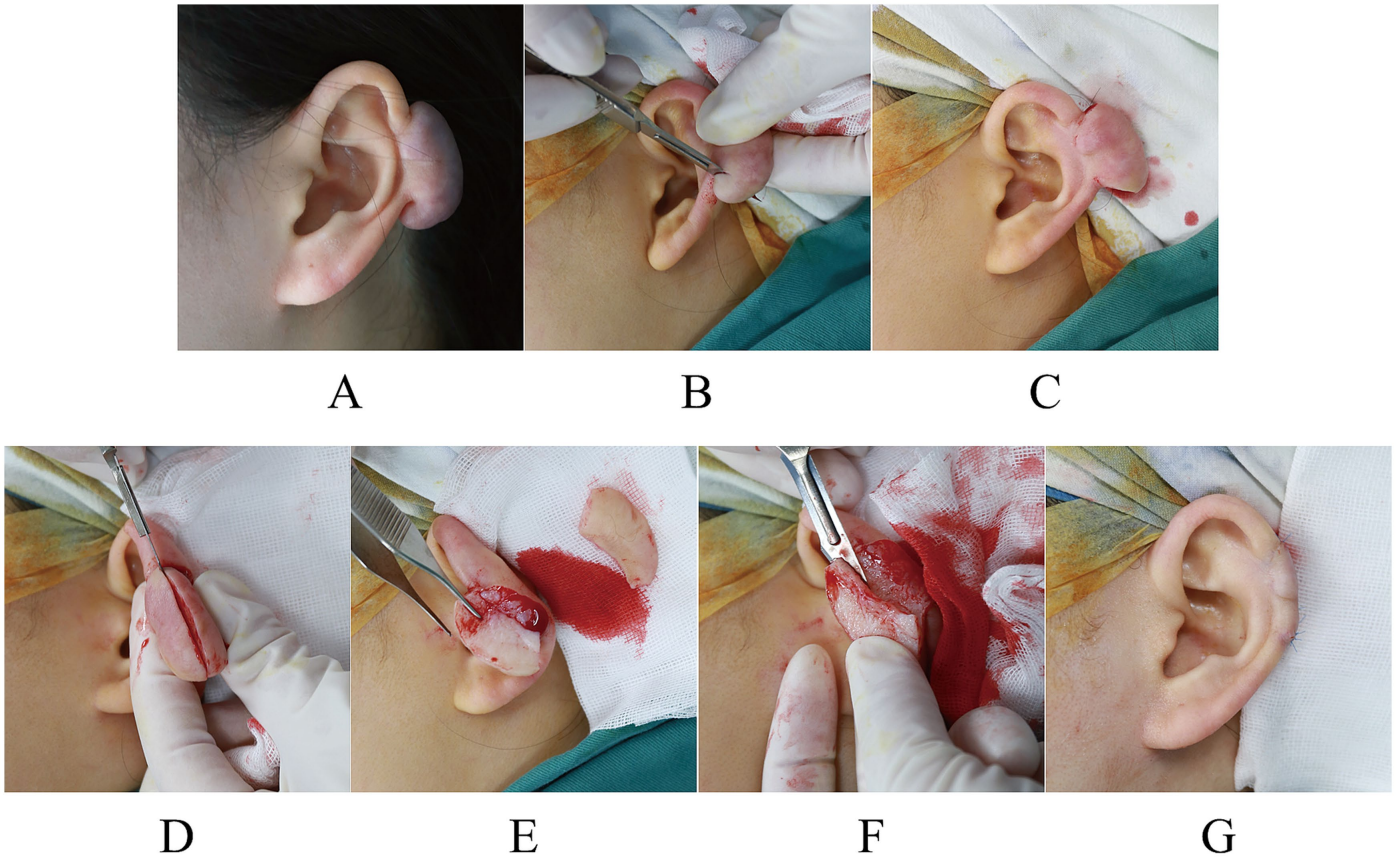
**(A)** Preoperative evaluation of a 23-year-old woman with a keloid on her left ear. **(B–F)** Course of the first to fifth blade core excision. **(G)** Postoperative.

**Figure 3:**
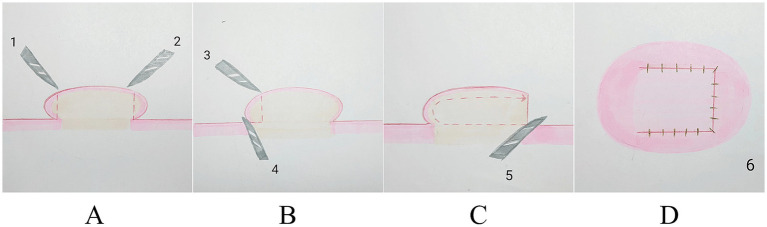
**(A–D)** Schematic diagram illustrates the process of core excision with five blades.

The initial and subsequent surgeries entailed excision of the keloid tissue that extended bilaterally from the keloid. The keloid can be approached using an 11-blade in an upward motion from its base (the preoperative skin lesions are shown in Figures 1A and 2A; reverse resection is also a viable option; Figures 1B,C, 2B,C, 3A).

The third excision is made to precisely match the size and shape of the keloid, with a longitudinal cut extending from the base of the keloid upward in parallel with the surface of normal skin (Figures 1D, 2D, 3B).

The fourth step involves the parallel removal of excessive scar tissue, aligning it with the surface of the surrounding healthy skin (Figures 1E,F, 2E, 3B).

The fifth incision should be made with the left index finger or the middle finger positioned close to the remaining keloid epidermis. The blade should follow along the medial nucleus basal plate of the keloid to remove its core, ensuring a thickness of the skin of approximately 1.5–2 mm (Figures 1G, 2F, 3C).

Finally, the flap’s edges were meticulously trimmed, and the wound was closed using a 6-0 (0.7 metric) blue monofilament polypropylene thread (Surgipro II, Covidien, Dublin, Ireland; Figures 1H, 2G, 3D). The wound was covered using sterile petroleum gauze and a dry white gauze dressing.

### 2.3 Postoperative adjuvant therapy

The patients underwent superficial electron beam radiotherapy within 24 h postoperatively. Subsequently, local dressing changes and the application of pressure dressings were performed following irradiation. The wound was irradiated consecutively for 5 days, with each session delivering a dose of 4 Gy (18). Consequently, the cumulative radiation dosage reached 20 Gy. The irradiation was precisely targeted to penetrate the surgical site at a depth ranging from 0.5 to 1 cm, with a radius of approximately 1 cm surrounding the surgical area. The patient and surrounding tissues and organs were effectively shielded from radiation using a lead barrier. The patient was administered a 0.5-cm wax mold by a radiologist to enhance the surface dose.

Intralesional corticosteroid injection was administered at the surgical site immediately postoperatively. No supplementary corticosteroids were utilized during follow-up. Two cases demonstrating mild recurrence required three supplemental corticosteroid injections during the surveillance period.

The sutures were ultimately extracted after 7 days. After the removal procedure, it is recommended to utilize a swimming nose clip for local pressure application in order to prevent recurrence for a period of 6 months. However, partial non-adherence occurred among patients reporting favorable postoperative recovery with the absence of pruritus, pain, or keloid hyperplasia. These patients discontinued compression application after 3 months and forwent scheduled clinic visits.

### 2.4 Questionnaires

Treatment outcomes were assessed using the Vancouver Scar Scale (VSS) and the Patient and Observer Scar Assessment Scale (POSAS) preoperatively and postoperatively. The postoperative VSS and POSAS results were evaluated within a time frame of 20 months.

### 2.5 Statistical analysis

Data processing and statistical analysis were conducted using SPSS 26.0 software. The outcome variables (each component of VSS and PSAS, OSAS, and POSAS) were not normally distributed. Represented by M [P25, P75], the outcome variables were analyzed using the Wilcoxon signed-rank test for paired comparison before and after interventions. Z score and two-sided *p*-values were reported, and lower scores indicated lighter scars, that is, better conditions.

## 3 Results

In this study, 11 patients, comprising 10 female and 1 male patients, presented with a total of 21 keloids. The mean age was 24.36 years (18–44 years). Two patients (with a total of three keloids) experienced recurrence despite the absence of postoperative superficial electron beam radiotherapy in previous medical facilities. An additional 9 patients (with a total of 18 lesions) underwent the procedure for the first time. Among the 21 keloid sites, 5 keloids were on the left ear and 16 keloids were on the right ear. The etiology was caused by ear puncture in 10 cases and trauma in 1 case. The patient history ranged from 1 to 8 years, with a mean age of 1.33 years. Table 1 shows demographic data for the 11 patients. The outcome of ear keloid treatment using five-blade core excision was favorable post-surgery, with a follow-up period of 20 months.

**Table 1:** Demographic data of the study group (11 patients).

Patient no.	Sex	Age (years)	Cause	Course (years)	History	Side of ear keloid and numbers
1	F	22	Ear piercing	1	First	Right/2
2	M	22	Ear piercing	1	First	Right/2 Left/2
3	M	32	Trauma	1	First	Right/1
4	F	19	Ear piercing	3	First	Right/2
5	F	22	Ear piercing	3	First	Right/2 Left/1
6	F	44	Ear piercing	3	Recurrence	Right/1
7	F	18	Ear piercing	1	First	Right/2
8	F	24	Ear piercing	2	First	Right/2
9	F	23	Ear piercing	8	First	Right/1
10	F	21	Ear piercing	2	First	Right/1
11	F	21	Ear piercing	3	Recurrence	Left/2

Among them, one patient had a total of four lesions on both sides of the ear, with two recurring at the edges of the left and right ears, respectively, while the other two did not recur on either earlobe. At the recurrence sites, the local tissue presented with firm consistency, subtle elevation, and a thickness of 1–2 mm. One patient with recurrent therapy was rejected for radiotherapy, while the remaining nine patients remained free from relapse.

The patients were assessed for VSS scores (Table 2) and POSAS scores (Table 3), both before and after treatment, with a follow-up duration of 20 months.

**Table 2:** Vancouver Scar Scale (VSS).

VSS	Before intervention	After intervention	*Z*	*p*-value
Pigmentation	2.00 (1.00, 3.00)	0.00 (0.00, 1.00)	−3.681	<0.01
Height	3.00 (2.00, 3.00)	0.00 (0.00, 0.00)	−3.862	<0.01
Vascularity	2.00 (1.00, 3.00)	0.00 (0.00, 1.00)	−3.685	<0.01
Pliability	3.00 (3.00, 3.00)	0.00 (0.00, 0.00)	−4.146	<0.01

**Table 3:** Patient and Observer Scar Assessment Scale (POSAS).

POSAS	Before intervention	After intervention	*Z*	*p*-value
PSAS	26.00 (24.00, 33.00)	2.00 (0.00, 5.50)	−4.017	<0.01
OSAS	32.00 (28.50, 37.00)	2.00 (1.00, 6.00)	−4.016	<0.01
POSAS	60.00 (50.50, 68.50)	3.00 (2.00, 7.00)	−4.015	<0.01

Compared with pre-intervention, the scores of all four dimensions of VSS decreased significantly after intervention (*p* < 0.01). The results showed that abnormal pigment, congestion, thickening, and stiffness of scars were significantly improved after intervention, and many indicators were concentrated at 0 points after the operation, suggesting that the clinical effect was significant.

Compared with pre-intervention, all POSAS indicators decreased significantly after intervention (*p* < 0.01), as determined by the Wilcoxon signed-rank test. This finding indicates that both subjective symptoms and objective appearance improved statistically and clinically after intervention.

## 4 Discussion

Keloid formation on the ear is a prevalent occurrence, with ear piercing being the primary instigating factor, particularly among adolescents and young adults (8, 10–12). The itching and pain caused by ear keloids not only bring significant discomfort to patients but also give rise to a range of psychological pressures due to their location on the exposed part of the face (19). Therefore, the treatment of ear keloid should involve the removal of keloid tissue and the restoration of the ear’s normal shape to minimize changes in appearance and other related aspects.

Currently, there is a lack of standardized classification for ear keloids, with only the Change-Park classification (based on keloid morphology and location) and anatomical location being mentioned in relevant literature. The two scholars categorized them based on the selection of therapeutic approaches.

The treatment of ear keloids can broadly be categorized into two main approaches: surgical interventions and non-surgical methods (such as cryotherapy, intralesional injections, laser therapy, and pressure therapy). Due to the short treatment duration, favorable outcomes, and improved postoperative esthetics, surgical intervention is frequently selected as the primary therapeutic approach for ear keloids.

The structure of the ear is intricate and exhibits strong stereoscopic characteristics in relation to the chest, limbs, and other body parts. The presentation of ear keloids encompasses a diverse range of forms: pedunculated, sessile, buried, distorted, and mixed forms (13, 14). Techniques for ear surgery include direct excision and suturing, core excision, various skin flaps, skin grafts, and dilators (11, 13, 15–17). Consequently, there is no universally optimal surgical plan for ear keloid treatment, as different individuals may opt for distinct approaches (20).

The procedure of keloidectomy involves excising the keloid core while preserving a portion of the surrounding keloid tissue to create a flap for defect repair. The structure of the auricle remains unchanged following a core incision, and due to its abundant blood supply, this surgical technique is commonly used for treating ear keloids (21, 22).

There are several principles for ear core excision:

1. The thickness of the flap is approximately 1–2 mm, which cannot be excessively thin or thick.
2. The removal of the keloid core should be minimized, and the resection should be performed concurrently with adjustment.
3. It is crucial not to damage the ear cartilage. The incision design should be aligned parallel to the long axis of the ear to maintain the curved contour of the earlobe and ear.
4. The surrounding normal skin tissue should remain undamaged.

Prasad et al. report that the surgical approach for treating ear keloids presents certain challenges: challenging tissue handling due to the small operation, dissection is time-consuming, and the base of the tissue is tough to clear completely (23).

Using five-blade core excision, the ear keloid should also adhere to the above principles; it is suitable for relatively independent keloids without significant deformity of the earlobe or auricle. Its advantages include the ability to quickly remove excess scar tissue from the surrounding area and base, resulting in relatively smooth skin grafts and shorter surgical times. The disadvantage is that the surgeon must concentrate and pay close attention to tactile sensations to avoid damaging or severing the skin graft.

For experienced surgeons, the skin thickness can be well controlled at 1–2 mm. However, for less experienced surgeons, it may lead to the following disadvantages:

1. The thickness of the skin flap cannot be well controlled at 1–2 mm.
2. If the finer feels poor, the flap may be sliced or the skin piece will be opened “skylight.”

Five-blade core excision method is suitable for treating keloids of the auricle and earlobe while preserving their original shape. We can conceptualize a keloid as a three-dimensional cube. The excess keloid skin on three sides were resected while preserving the top and one side of the integument. Currently, the tongue-shaped flap is a relatively common method for ear keloid excision in China. This technique requires the preoperative design of the flap shape to avoid the excessive removal of keloid tissue and achieve tension-free wound closure. Consequently, the preliminary design demands significant experience from the surgeon. In contrast, the five-blade core excision technique first involves removing excess tissue at the base of the protruding keloid, which imposes relatively lower experience requirements on the surgeon. Second, the tongue-shaped flap excises scar tissue in layers to prevent over-resection, which could lead to localized depressions and compromise natural thickness and esthetics (15). The five-blade core excision involves flat shaving along the base, eliminating the need for repeated layered excision, thereby saving surgical time. Its key step is to pay attention to tactile feedback when excising the part connecting the base to the flap, avoiding detachment from the ear tissue and the formation of a free skin graft.

Lawrence et al. revealed that the recurrence rate of ear keloids was not found to be associated with factors such as piercing length, gender, keloid size, or keloid occurrence time (24, 25). Li et al. found that the postoperative recurrence of ear keloid often occurred from 2 weeks to 1 month after surgery. The recurrence rate of the extensive ear keloids is much higher than that of other ear keloids. In surgical method selection, the flap’s recurrence rate is comparable to that of direct suturing; thus, there is no significant concern regarding an increased recurrence rate due to residual fibrous tissue from the ear keloid flap (16).

Aseptic techniques, minimally invasive approaches, precise surgical resection and suturing, tension-free wound closure, meticulous hemostasis, and prompt postoperative follow-up are crucial for achieving success in preventing and reducing recurrence (19, 26, 27).

After the surgical procedure, an effective pressure dressing can optimize the fit of the skin flap to the wound and mitigate the risk of skin flap hematoma. Nason et al. reported that the recurrence rate of keloid scar decreased from 67 to 18% using pressure therapy (9, 28–30). The exact mechanism remains unclear. It may attributed to a reduction in blood inflow, subsequently diminishing the relevant factors that promote keloid growth and effectively inhibiting keloid recurrence (31). The application of excessive pressure should be avoided during the postoperative wound healing process to prevent ischemic necrosis of the skin (31).

The administration of radiotherapy can effectively attenuate local angiogenesis, impede fibroblast proliferation, impair cellular functionality, and significantly diminish the incidence of postoperative recurrence (22).

In this study of 11 patients, one patient exhibited minor epidermal erosion postoperatively. Considering the excessive duration of postoperative compression, the erosive area successfully healed following the discontinuation of compression. We subsequently modified the protocol to apply compression for 8 h daily for the first 7 days postoperatively, followed by 24-h compression after suture removal. No erosion was observed in the patient. One patient refused radiotherapy and experienced recurrence, indicating that radiotherapy is a key factor in controlling ear scar tissue. Therefore, postoperative radiotherapy should be considered a first-line preventive measure against keloid recurrence. One patient had four keloids, two of which recurred at the ear margin and two did not. The two keloids spanning the ear rims recurred, whereas the other two located on the auricles did not. This discrepancy is likely attributable to the three-dimensional structure of keloids that span the ear, which involves both anterior and posterior surfaces. Whether anterior or posterior fixed-field radiotherapy was administered, minor variations in the depth of certain areas of the keloids may have resulted in dose inhomogeneity, ultimately leading to recurrence. We will further discuss this finding with the radiotherapy department. Other patients showed no recurrence, infection, hyperpigmentation, radiation dermatitis, or other complications during the follow-up period and were satisfied with the results.

## 5 Limitations

This study has some limitations. It had a small sample size and a follow-up period of less than 3 years. More patients and a longer follow-up period of up to 5 years may show the long-term effects of the therapy. This surgical method is not suitable for keloids that surround the ear or have lost their ear shape.

## 6 Conclusion

This study provides preliminary evidence of the feasibility and efficacy of the core excision five-step technique in the surgical treatment of ear keloids, but further controlled trials and expanded case studies are required for additional validation.

## Data Availability

The original contributions presented in the study are included in the article/supplementary material, further inquiries can be directed to the corresponding authors.
